# Secondary Metabolic Profiles of Two Cultivars of *Piper nigrum* (Black Pepper) Resulting from Infection by *Fusarium solani* f. sp. *piperis*

**DOI:** 10.3390/ijms18122434

**Published:** 2017-12-07

**Authors:** Shirlley F. M. da Luz, Lydia F. Yamaguchi, Massuo J. Kato, Oriel F. de Lemos, Luciana P. Xavier, José Guilherme S. Maia, Alessandra de R. Ramos, William N. Setzer, Joyce Kelly do R. da Silva

**Affiliations:** 1 Programa de Pós-Graduação em Biotecnologia, Universidade Federal do Pará, Belém 66075-900, Brazil; shirlleyluz@ufpa.br (S.F.M.d.L.); luxavier@gmail.com (L.P.X.); 2 Laboratório de Química de Produtos Naturais, Instituto de Química, Universidade de São Paulo, São Paulo 05508-000, Brazil; lydyama@gmail.com (L.F.Y.); majokato@iq.usp.br (M.J.K.); 3 Centro de Pesquisa Agroflorestal da Amazônia Oriental, Empresa Brasileira de Pesquisa Agropecuária, Belém 66095-903, Brazil; oriel.lemos@embrapa.com; 4 Programa de Pós-Graduação em Recursos Naturais da Amazônia, Universidade Federal do Oeste do Pará, Santarém 68035-110, Brazil; gmail@ufpa.br; 5 Instituto de Estudos em Saúde e Biológicas, Universidade Federal do Sul e Sudeste do Pará, Marabá 68501-970, Brazil; rezende@unifesspa.edu.br; 6 Department of Chemistry, University of Alabama in Huntsville, Huntsville, AL 35899, USA; setzerw@uah.edu

**Keywords:** plant-pathogen interaction, essential oils, sesquiterpenes, α-bisabolol, δ-elemene, alkamides

## Abstract

Bragantina and Cingapura are the main black pepper (*Piper nigrum* L.) cultivars and the Pará state is the largest producer in Brazil with about 90% of national production, representing the third largest production in the world. The infection of *Fusarium solani* f. sp. *piperis*, the causal agent of *Fusarium* disease in black pepper, was monitored on the cultivars Bragantina (susceptible) and Cingapura (tolerant), during 45 days’ post infection (dpi). Gas Chromatography-Mass spectrometry (GC-MS) analysis of the volatile concentrates of both cultivars showed that the Bragantina responded with the production of higher contents of α-bisabolol at 21 dpi and a decrease of elemol, mostly at 30 dpi; while Cingapura displayed an decrease of δ-elemene production, except at 15 dpi. The phenolic content determined by the Folin Ciocalteu method showed an increase in the leaves of plants inoculated at 7 dpi (Bragantina) and 7–15 dpi (Cingapura); in the roots, the infection caused a phenolic content decrease in Bragantina cultivar at 45 dpi and an increase in the Cingapura cultivar at 15, 30 and 45 dpi. High Performance Liquid Chromatography-Mass spectrometry (HPLC-MS) analysis of the root extracts showed a qualitative variation of alkamides during infection. The results indicated that there is a possible relationship between secondary metabolites and tolerance against phytopathogens.

## 1. Introduction

Black pepper (*Piper nigrum* L.) is by far one of the most important spices and has been used for centuries in food, to treat many ailments, and as cosmeceutical products. It is cultivated in Asian countries such as Malaysia, India, Indonesia, Thailand, Vietnam, China and Sri Lanka, in addition to Madagascar and Brazil [1,2]. In Brazil, the cultivation of *P. nigrum* started in the 17th century and since 1933, it has been economically explored by Japanese immigrants [3]. Pará state is the largest producer in Brazil, with about 90% of national production, representing the third largest production in the world. Bragantina and Cingapura are the main black pepper cultivars in Brazil. The Bragantina cultivar is a hybrid obtained from South India, also known as “Panniyur”, exhibiting large leaves, heart-shaped and long spikes with an approximate length of 14.0 cm. The Cingapura cultivar, also known as “Kuching”, has small and narrow leaves, short spikes on average of 7.0 cm length. The average production per plant is 3 kg and 2.5 kg of black pepper for Bragantina and Cingapura cultivars, respectively. The amount of the amide piperine, the pungent principle of black pepper, is higher in the fruits of Cingapura (69.1%) cultivar than Bragantina (41.6%) cultivar [4,5].

The phytopathogens *Phytophthora capsici* (India) and *Fusarium solani* f. sp. *piperis* (Brazil) are the causative agents of root rot or *Fusarium* disease (fusariosis) to the crop of black pepper [6]. The first symptoms of fusariosis start at the roots or branches and promote falling leaves, root rot, and plant death [3]. Pathogens that cause wilting, such as *Fusarium solani* f. sp. *piperis*, have a hemibiotrophic life cycle. This and other similar pathogens initially colonize living tissue and, at a later stage, colonize dead tissue [7].

Despite the studies for controlling fusariosis in black pepper, there are no reports on secondary metabolites involved in this plant-pathogen interaction. In general, the plant presents specific defense mechanisms to respond to the attack of pathogens and herbivores, which include the mediation of terpenoid and polyphenolic compounds in the biologic systems [8]. Metabolomics provides a means for comparison between different species, and it can assist in identification of key genes and enzymes involved in various physiological processes, such as biotic and abiotic stresses [9]. The approach is emerging as a promising means of development of biomarkers for the early diagnosis of diseases, interaction mechanism, identification of resistance and susceptibility in various cultures [10]. For example, the infection of mango (*Mangifera indica*) by *Fusarium moniliforme* var. *subglutinans* has increased the production of phenolic compounds [11]; and the volatile compounds of wheat (*Triticum aestivum*) have been mentioned as the specific biomarkers involved in resistance against *Fusarium graminearum* infection [12]. Study of metabolomic profiles involved in host-pathogen interaction has revealed significant differences in the composition of secondary metabolites in leaves and roots, indicating that these organs employ distinct chemical defense systems [13]. The analysis by Gas Chromatography-Mass Spectrometry (GC-MS) of the volatile compounds emitted in the interaction between eucalyptus (*Eucalyptus globulus*) and the pathogen *Teratosphaeria nubilosa* identified about 40 biomarkers that can diagnose the disease before the observation of symptoms [14].

The understanding of these functions in the defense system of plants is important for obtaining resistant cultivars by biotechnological breeding [15]. Thus, the aim of this study was to identify the volatile constituents produced by the cultivars of Bragantina and Cingapura, during infection by *F. solani* f. sp. *piperis*, in order to contribute to the discovery of potentially useful secondary metabolites in the black pepper defense system.

## 2. Results

### 2.1. Evaluation of Susceptibility and Tolerance Of Black Pepper Cultivars after Inoculation with Fusarium solani f. sp. piperis

**Figure 1:**
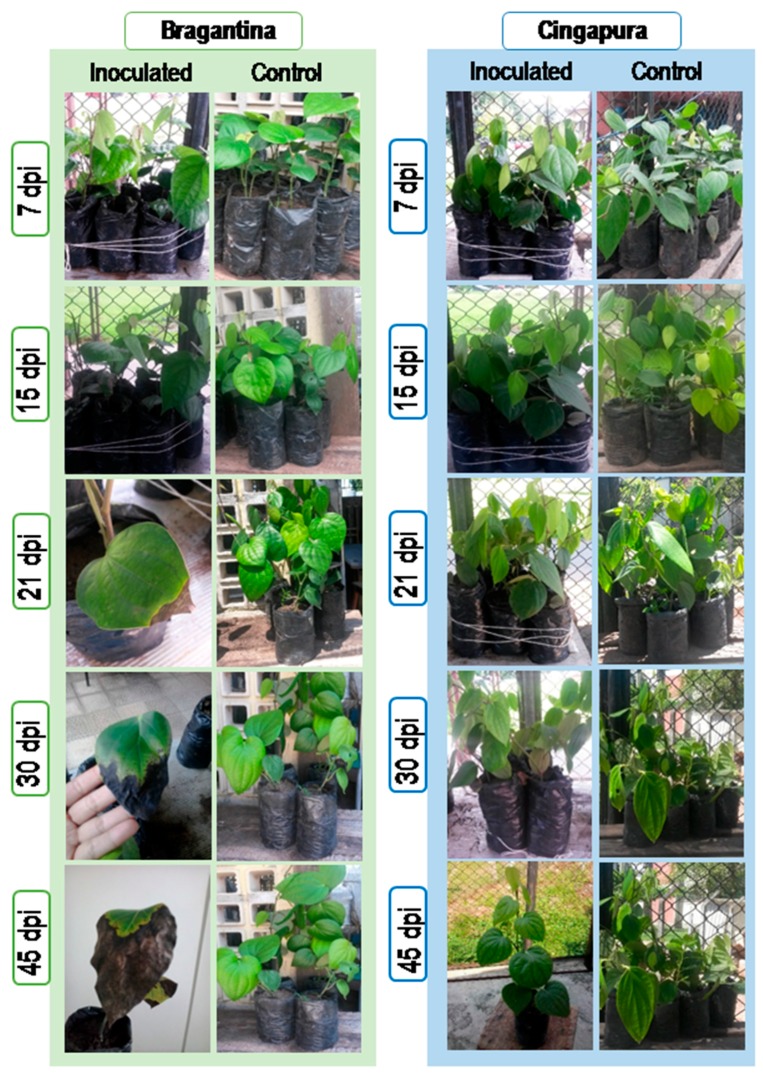
Visual evaluation of Fusariosis symptoms in *P. nigrum* cultivars during inoculation by *F. solani* f. sp. *piperis*. dpi: days post infection.

Progression of the host-pathogen interaction was monitored visually in the periods of 7, 15, 21, 30 and 45 days’ post infection (dpi) (Figure 1). For both cultivars inoculated, no visible foliar symptoms were observed at 7 and 15 dpi. The first symptoms of *Fusarium* disease were observed at 21 dpi, when the leaves turned yellow in the susceptible Bragantina cultivar (Figure 1, 21 dpi). Disease symptoms progressed rapidly in the Bragantina cultivar, with the partial necrosis of leaves and the total drying of branches (Figure 1, 30 and 45 dpi). On the other hand, the plants of Cingapura cultivar did not display foliar symptoms at all during the 45-day period.

### 2.2. Profiles of Volatile Compounds of the Black Pepper Cultivars

The *P. nigrum* leaves, from the Bragantina and Cingapura cultivars, after *Fusarium* infection, were submitted to simultaneous distillation and extraction to obtain their volatile concentrates. Individual components were identified by comparison of both mass spectrum and gas chromatography (GC) retention data with authentic compounds, previously analyzed and stored in the data system. Commercial libraries, which contained retention indices and mass spectra of volatile compounds commonly found in essential oils were used [16,17]. The GC and GC-MS analysis of the volatile concentrates from inoculated plants (IP) and control plants (CP) allowed the identification of 30 constituents in Bragantina cultivar and 54 components in Cingapura cultivar (Table 1 and Table 2). In the volatile concentrates of Bragantina cultivar, the identified constituents presented an average of 91.6%, with the predominance of oxygenated sesquiterpenes (IP, 77.4%; CP, 82.0%), where α-bisabolol (IP, 56.3%; CP, 51.5%), elemol (IP, 12.6%; CP, 24.0%) were the main compounds, followed by a smaller amount of germacrene D (IP, 3.6%; CP, 4.4%), a sesquiterpene hydrocarbon. For volatile concentrates of Cingapura cultivar the identified constituents showed a median composition of 95.2%, with prevalence of sesquiterpene hydrocarbons (IP, 76.2%; CP, 72.6%), where δ-elemene (IP, 57.1%; CP, 53.3%), α-zingiberene (IP, 4.3%; CP, 5.1%) and β-caryophyllene (IP, 4.2%; CP, 4.7%) were the major components, followed by muurola-4,10(14)-dien-1-β-ol (IP, 4.1%; CP, 4.9%), an oxygenated sesquiterpene.

### 2.3. Variation in Volatile Profiles of the Black Pepper Cultivars, after Fusarium Infection

**Figure 2:**
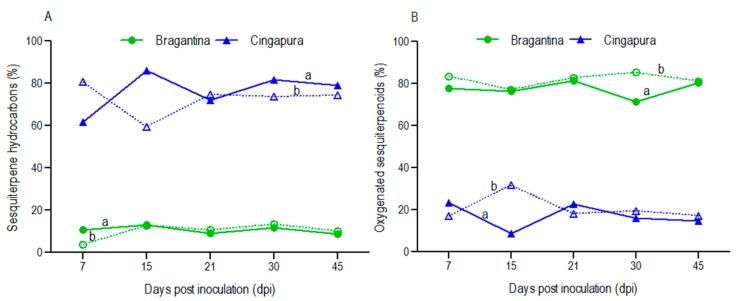
Sesquiterpenes variation in *P. nigrum* cultivars during infection by *F. solani* f. sp. *piperis*. (**A**) Sesquiterpenes hydrocarbons; (**B**) Oxygenated sesquiterpenes; (a) inoculated plants; (b) control plants. dpi: days post infection.

The volatile concentrates of Bragantina cultivar were dominated by oxygenated sesquiterpenes (IP, 77.4%; CP, 82.0%), followed by sesquiterpene hydrocarbons (IP, 10.7%; CP, 10.2%) as minor compounds (Table 1, Figure 2). The production of oxygenated sesquiterpenes showed a drastic decrease (71.5%) in the inoculated plants, just at 30 dpi, which can be associated with the progress of *Fusarium* disease during that cultivation period. In contrast, the Cingapura cultivar showed sesquiterpene hydrocarbons as main compounds (IP, 76.2%; CP, 72.6%) and oxygenated sesquiterpenes in minor proportions (IP, 17.1%; CP, 20.7%) (Table 2, Figure 2). An increase in production of sesquiterpene hydrocarbons was observed with inoculated plants at 15, 30 and 45 dpi. On the other hand, the sesquiterpene hydrocarbons content was equivalent in the inoculated and control plants at the 21 dpi.

Inoculated plants (IP) of Bragantina cultivar showed quantitative difference for α-bisabolol and elemol, the main compounds, when compared to control plants (CP) for the duration of cultivation (from 7 to 45 dpi). The production of α-bisabolol was higher at 21 dpi (IP, 64.3%; CP, 51.4%), 30 dpi (IP, 60.1%; CP, 49.8%), and 45 dpi (IP, 60.7%; CP, 54.6%). With respect to elemol, however, the situation was reversed. There was a reduction of its percentage in the inoculated plants when compared to the control plants: 7 dpi (IP, 19.0%; CP, 27.3%), 21 dpi (IP, 15.3%; CP, 28.4%), 30 dpi (IP, 0.7%; CP, 32.9%), and 45 dpi (IP, 3.4%; CP, 9.2%). The content of minor compounds also showed significant variation in the inoculated plant, after the appearance of the symptoms of *Fusarium* disease. For example, increase of β-bisabolene and *E*-β-ocimene, decrease of germacrene D, variation for the isomers β-, γ- and δ-elemene, as well as a large production of β-eudesmol at 30 dpi (10.7%) and 45 dpi (14.9%). Other alterations can also be observed in the inoculated Bragantina cultivar. *E*-β-Ocimene (1.2%), β-caryophyllene (1.0%), germacrene D (4.2%), β-bisabolene (3.6%) (2*Z*,6*Z*)-farnesol (1.6%) and (2*Z*,6*E*)-farnesol (3.2%) increased above 1%.

Similarly, inoculated plants of Cingapura cultivar also showed qualitative and quantitative differences in the volatile profiles, due to infection, particularly for δ-elemene and β-caryophyllene, their main compounds. δ-Elemene production was higher at 15 dpi (IP, 73.6%; CP, 48.7%), 30 dpi (IP, 61.2%; CP, 57.1%) and 45 dpi (IP, 55.8%; CP, 48.1%). However, β-caryophyllene increased only at 30 dpi (IP, 3.9%; CP, 3.0%) and 45 dpi (IP, 5.4%; CP, 4.9%). The sesquiterpenes α-ylangene (1.2%), β-atlantol (3.4%), 1,10-di-*epi*-cubebol (1.6%) and eudesma-4,11-dien-2α-ol (1.2%) were produced above 1%, only by inoculated plants at 7 dpi. *E*-Nerolidol showed a decrease at 7 dpi (IP, 2.7%; CP, 4.8%), 15 dpi (IP, 1.3%; CP, 8.5%), 21 dpi (IP, 3.9%; CP, 6.6%) and 30 dpi (IP, 2.7%; CP, 4.0%).

### 2.4. Variation in Total Phenolic Compounds Profiles of the Black Pepper Cultivars, after the Fusarium Infection

**Figure 3:**
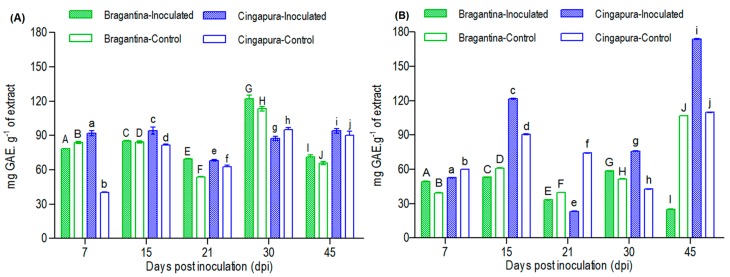
Phenolic content variation in *P. nigrum* cultivars during infection by *F. solani* f. sp. *piperis*. (**A**) Leaves (**B**) Roots, mg GAE·g^−1^ (mg of gallic acid equivalent per gram of extract). A, a, B, b, C, c, D, d, E, e, F, f, G, g, H, h, I, i, J, j values with different letters are statistically different at the *p* < 0.05 level (Tukey’s test).

The variation in total phenolic compounds was evaluated in the leaves and roots of Bragantina and Cingapura cultivars, after the inoculation, and expressed as milligrams of gallic acid equivalents per gram of gram of extract (mg GAE·g^−1^). Bragantina cultivar leaves showed no significant variation, while the Cingapura cultivar presented a significant production in the inoculated plants at 7 dpi, which can be associated as a response to the infection in the early days (Figure 3). Root total phenolic compounds showed different behavior in comparison to the leaves. In the Bragantina cultivar (susceptible to *Fusarium*) the only significant change was observed at 45 dpi, which was a dramatic decrease of total phenolic compounds in the inoculated plants, when compared to control plants (IP, 39.9 mg GAE·g^−1^; CP, 91.9 mg GAE·g^−1^). On the other hand, Cingapura cultivar (tolerant to *Fusarium*) produced higher content of total phenolic compounds in the inoculated plants at 15 (IP, 91.9 mg GAE·g^−1^; CP, 39.9 mg GAE·g^−1^), 30 (IP, 75.8 mg GAE·g^−1^; CP, 42.6 mg GAE·g^−1^) and 45 dpi (IP, 173.8 mg GAE·g^−1^; CP, 108.7 mg GAE·g^−1^) (Figure 3). Conversely, there was a decrease in total phenolic compounds at 21 dpi (IP, 23.2 mg GAE·g^−1^; CP, 74.1 mg GAE·g^−1^), which could be related to the beginning of the development cycle of *Fusarium*, since the first symptoms for the susceptible cultivars were observed in this period.

### 2.5. Chemical Profiles of the Root Extracts from Bragantina and Cingapura Cultivars, after Fusarium Infection

**Figure 4:**
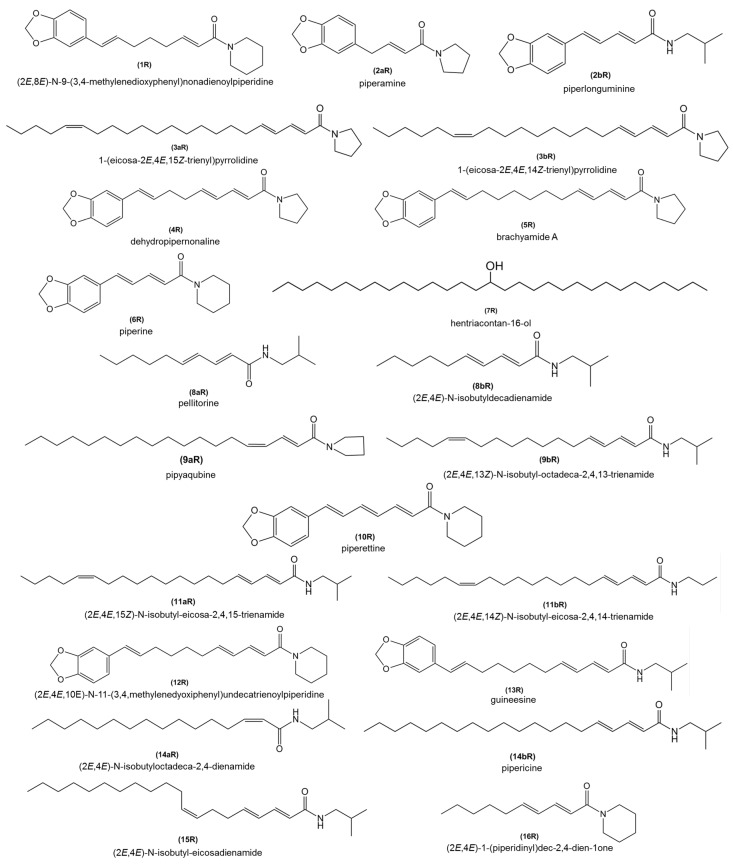
Identified compounds in roots of *P. nigrum* cv. Bragantina and Cingapura during infection with *F. solani* f. sp. *piperis*.

High Performance Liquid Chromatography-Mass Spectrometry (HPLC-MS) analysis of root extracts of Bragantina and Cingapura cultivars resulted in the identification of various alkamide compounds (Figure 4). Individual compounds were identified by comparison of their mass spectra with those previously reported for *P. nigrum* samples. The alkamides compounds, such as piperine (6R), pellitorine/(2*E*,4*E*)-*N*-isobutyldecadienamide (8aR/8bR), pipyaqubine/(2*E*,4*E*,13Z)-*N*-isobutyl-octadeca-2,4,13-trienamide (9aR/9bR), (2*E*,4*E*)-*N*-isobutyloctadeca-2,4-dienamide/pipericine (14aR/14bR), (2*E*,4*E*,10*E*)-*N*-11-(3,4,methylenedyoxiphenyl)undecatrienoylpiperidine 12R and guineesine (13R) were identified in both cultivars. The inoculated Bragantina cultivar produced the compounds piperamine/piperlonguminine (2aR/2bR) and pipyaqubine/(2*E*,4*E*,13*Z*) -*N*-isobutyl-octadeca-2,4,13-trienamide (9aR/9bR) only at 7 dpi and 15 dpi. Higher qualitative difference can be observed at 21 dpi, in which the alkamides (2*E*,8*E*)-*N*-9-(3,4-methylenedioxyphenyl)nonadienoylpiperidine (1*R*), 1-(eicosa-2*E*,4*E*,15*Z*-trienyl)pyrrolidine/1-(eicosa-2*E*,4*E*,14*Z*-trienyl)pyrrolidine (3aR/3bR), pipyaqubine/(2*E*,4*E*,13*Z*)-*N*-isobutyl-octadeca-2,4,13-trienamide (9aR/9bR) and (2*E*,4*E*)-*N*-isobutyl-eicosadienamide (15*R*) were absent. The chemical profile of Cingapura cultivar did not display qualitative changes, which could be attributed to its tolerance. The compounds (2*E*,4*E*)-*N*-isobutyloctadeca-2,4-dienamide/pipericine (14aR/14bR) and pipyaqubine/(2*E*,4*E*,13*Z*) -*N*-isobutyl-octadeca-2,4,13-trienamide (9aR/9bR) were identified in the inoculated plants at 7 dpi and 15 dpi, respectively (Table 3 and Table 4).

### 2.6. PCA Analysis

**Figure 5:**
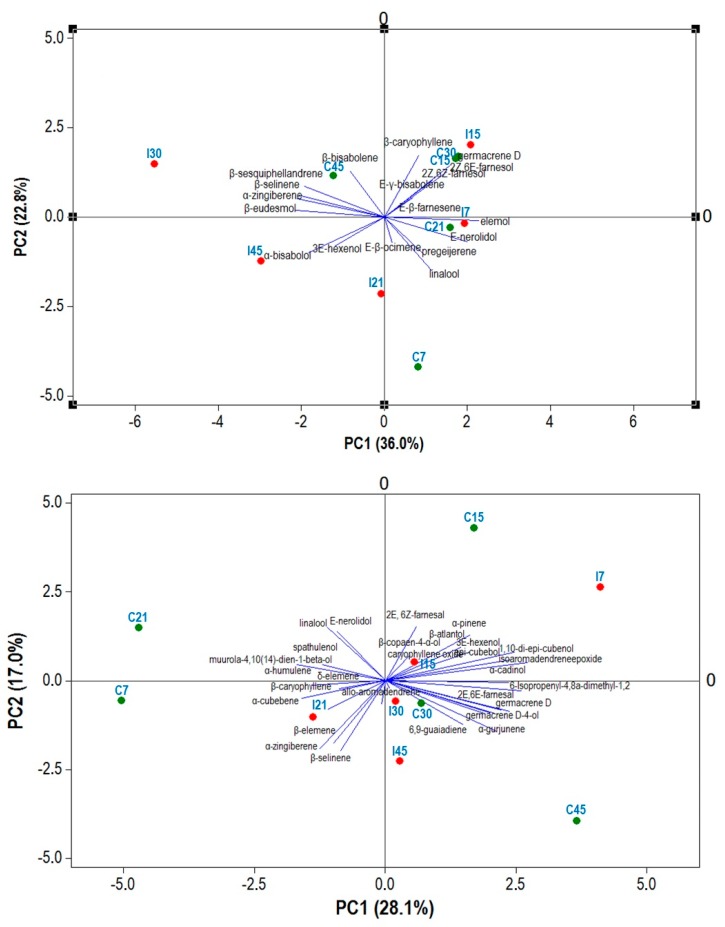
Bidimensional plot of first two components obtained by Principal Component Analysis (PCA) of *P. nigrum* cultivars based on Essential Oil (EO) chemical composition. Inoculated plants: I7, I15, I21, I30 and I45. Control Plants: C7, C15, C21, C30 and C45. (**A**) Bragantina; and (**B**) Cingapura. PC1: First Principal Component, PC2: Second Principal Component.

The components first Principal Component (PC1) and second Principal Component (PC2). displayed the patterns found in Bragantina (58.8%) and Cingapura (45.2%) cultivars. Bragantina cultivar showed a positive PC1 correlation mostly with elemol (0.351), *E*-nerolidol (0.307), germacrene D (0.243) and (2*Z*,6*E*)-farnesol (0.232) and its including the samples C7, C15, C21, C30, I7 and I15. Compounds with more positive loadings in PC2 were β-caryophyllene (0.417), germacrene D (0.347), β-bisabolene (0.313), (2*Z*,6*E*)-farnesol (0.307) and grouped the samples C15, C30, C45, I15 and I30 (Figure 5A). PC1 of the Cingapura cultivar showed higher positive correlation with isoaromadendrene epoxide (0.293), eudesma-4,11-dien-2α-ol (0.279), 1,10-di-*epi*-cubenol (0.256), germacrene D (0.255) and α-gurjunene (0.229). PC2 of the Cingapura cultivar presented a positive correlation with (2*E*,6*Z*)-farnesal (0.269), *E*-nerolidol (0.266), linalool (0.246), α-gurjunene (0.229) and α-pinene (0.228) (Figure 5B).

## 3. Discussion

For populations of an agricultural crop and its associated pathogens, it has been seen that the genetic variation for its resistance and infectivity is ubiquitous [18,19]. Although *P. nigrum* cultivars are not completely resistant to *Fusarium*, no contrary evidence has been reported yet [20].

The main compounds that make up the aroma of black pepper are α- and β-pinene, sabinene, myrcene, limonene and β-caryophyllene, followed by minute quantities of δ-3-carene, β-phellandrene, elemicin, muurolol, cubenol and bulsenol [21,22]. Normally, the *P. nigrum* cultivars have shown variability in their essential oil constituents [23,24,25]. For example, the main compounds identified in 26 different cultivars of black pepper collected in Kerala, India, were elemol and germacrene D in the leaf oils, and β-caryophyllene in the fruit oils [26].

The essential oil of Cingapura cultivar was dominated by sesquiterpenes hydrocarbons (61.8–86.1%), as δ-elemene (57.6%) and β-caryophyllene (3.8%), in contrast with a specimen collected in Cameroon that showed low concentrations of δ-elemene (2.6%) and β-caryophyllene (7.3%) [21]. Inoculated plants activate signal transduction cascades mediated by plant hormones, which lead to higher expression of genes related to defense [27]. There are many chemical compounds involved in interactions between plants, which include aldehydes and low molecular weight alcohols such as 2*E*-hexenal, 3*Z*-hexen-1-ol, terpenes such as myrcene and ocimene consisting of mixtures of *E*-β-ocimene, *Z*-β-ocimene and *allo*-ocimene [28].

The response to infections of Bragantina and Cingapura cultivars promotes the production of 3*E*-hexenol, which is considered a volatile produced in green leaves in response to a herbivore attack. Cingapura inoculated plants produced 3*E*-hexenol (7 and 15 dpi), which indicated a response in the early stages. However, in the Bragantina cultivar their emission was observed from 21 to 45 dpi only in inoculated plants. In the oxidation of linoleic acid by the action of lipoxygenase enzymes (LOX), 3*Z*-hexenal is the first product formed followed by isomerization reactions resulting in the formation of 2*E*-hexenal. These aldehydes are converted to the corresponding alcohols by dehydrogenase action [29,30].

Among minor compounds, *E*-β-ocimene was frequent and showed higher concentrations in Bragantina inoculated plants at 7, 21 and 45 dpi and Cingapura plants at 7 dpi. This compound has been reported as important in plant-plant interactions, pathogen resistance and changes in abiotic factors [31]. The emission of *E*-β-ocimene was correlated during interaction of tobacco and peanut, inoculated by *Pseudomonas syringae* and *Sclerotium rolfsii*, respectively [32,33,34,35,36]. The plants present direct and indirect defense responses when they are damaged by herbivores, or inoculated by fungal and bacterial pathogens [37]. The significant change in the production of α-bisabolol observed in Bragantina cultivar at 21 dpi and δ-elemene in Cingapura cultivar at 15 dpi may be a response to plant-pathogen interactions, since some sesquiterpenes are produced as phytoalexins by the plant defense system [38]. PCA analysis of volatile compounds from each cultivar discriminated the cultivar in two groups. For the Bragantina cultivar, the negative loadings in the PC1 were observed for inoculated samples after 21 dpi, such as I30, I45 and I21, except the sample C45. However, the groups in the Cingapura samples did not display a response after the infection.

Oxygenated sesquiterpenes were dominant in Bragantina cultivar (susceptible) and sesquiterpene hydrocarbons in Cingapura cultivar (tolerant). The Bragantina cultivar did not display a direct influence of oxygenated sesquiterpenes during the infection. In general, sesquiterpene levels decreased in inoculated plants as initial response to disease (from 7 to 15 dpi) and after 21 dpi, this difference decreased. However, in Cingapura cultivar, sesquiterpene hydrocarbons showed a direct correlation with infection, because a high production was observed in early stages (7–15 dpi). From 30 dpi, a decrease in this difference was observed (Figure 2). The increase in concentration of sesquiterpene hydrocarbons was determined as a marker of infection by *Fusarium* in corn. Inoculated grains produced high concentrations of β-selinene, α-selinene, β-macrocarpene, β-bisabolene and trichodiene during infection by *F. graminearum*, *F. verticillioides* and *F. subglutinans*, while uninoculated grains showed high levels of cycloisosativene and α-ylangene [39]. These results are in contrast with the earlier reports on response of two lines of *P. nigrum* inoculated by *Phytophthora capsici*, which the higher accumulation of phenolic compounds in the roots and stem were observed with the susceptible line (Sreekara) in comparison to the tolerant (04-P24) [40].

Plants submitted to stress displace the energy invested in primary metabolism to secondary metabolism and consequently there is a reduction in the growth rate, reproduction and competitiveness. Subsequently, the synthesis of secondary metabolites is altered for survival and results in regulation of carbon flow between primary and secondary metabolism [41]. Resistance to pests or diseases of certain species could be related to the concentration and variety of phenolic compounds [42,43]. Due to their toxicity, compounds such as flavonoids and hydroxycinnamic acids act as passive or inducing barriers against microorganisms and herbivores [43,44].

The infection by *Colletotrichum nymphaea*, the causal agent of anthracnose in strawberry (*Fragaria* sp.) in two cultivars “Elsanta” (susceptible) and “Honeoye” (resistant) promoted an increase in the concentration of phenolic compounds as ellagic acid derivatives, flavonols and flavan-3-ols [45]. Furthermore, multivariate analysis of phenolic compounds of different mango cultivars (*Mangifera indica*) allowed the identification of biomarkers in the groups as tolerant and susceptible to *Fusarium* infection. The larger levels of phenolic compounds were observed in more tolerant cultivars “Nam docmai” and “Roza” [46]. These results support the hypothesis that phenolic compounds play an important role in host-resistance in inoculated tissue during plant-pathogen interactions [43]. Despite the variation in total phenolic contents in the roots of both cultivars, they displayed a wide diversity of alkamides. Additionally, it is important to emphasize the low selectivity of phenolic compounds by Folin-Ciocalteu assay, once it displays false positives to non-phenolic reducing species, such as vitamin C, amino acids and thiol derivatives [47]. In addition, many nitrogen-containing compounds showed considerable reactivity toward Folin Ciocalteu reagent. These classes include hydrazines, hydroxylamines, guanidines, tertiary amines, aromatic amines, pyrroles, and indoles [48].

The plant with the highest levels of alkamides (AAs) are found in species of Asteraceae, Piperaceae, Solanaceae and Rutaceae [49,50]. There are numerous studies on antimicrobial activities of AAs by different test methods in vitro, e.g., dilution, disk diffusion and bioautography [51]. The antifungal activity of AAs has been reported against *Cladosporium* species [52,53]. In plants, these compounds play an important role in seedling development in physiological or stress conditions [54]. Kim et al. (2010) [55] described two hypothetical mechanisms of their protective role: (1) NAE (*N*-acylethanolamines) accumulation could modulate the level of other lipids (e.g., phosphatidic acid) in response to pathogens or (2) NAEs might interfere with the quorum sensing mechanism (i.e., inter-pathogen communication to coordinate their activities) of pathogen.

## 4. Materials and Methods

### 4.1. Plant Materials

Plants of black pepper, from Brangatina and Cingapura cultivars, were provided by Empresa Brasileira de Pesquisa Agropecuária (EMBRAPA)—Campus of Amazônia Oriental, city of Belém, state of Pará, Brazil. Twenty-eight plants of each cultivar, with three months of cultivation, were used in the experiment.

### 4.2. Fungal Strains and Inoculation on Black Pepper

The origin and identification of the fungal strain used to infection experiments are not clear, nor reference given. *F. solani* f. sp. *piperis* isolates were collected from black pepper commercial planting located in Baião city (Pará State). The fungal identification was performed based on morphological characteristics of conidia and microconidia in the inoculant solution, which present an oval shape and fusoid, respectively. The fungus was cultured on potato dextrose agar (PDA) for 10 days, at 27 °C, before starting the experiment. The fungi spores were collected in sterile water and the inoculant solution was prepared at a concentration of 1.43 × 10^5^ spores/mL. The roots of each plant of black pepper cultivars were inoculated with the spore solution according to Meireles, while plants of the control group were treated with distilled water [7]. The *Fusarium* infection and the production of secondary metabolites were monitored at 7, 15, 21, 30 and 45 days post inoculation (dpi). After the experimentation, the phytopathogen was again isolated from the roots of the black pepper cultivars under study.

### 4.3. Volatile Concentrates and Extracts Preparation

The volatile constituents were extracted from the leaves (3 g) of each treated plant by simultaneous distillation-extraction process using a Likens-Nickerson apparatus and n-pentane (3 mL) as solvent. After 2 h, the organic fraction was removed. Fresh leaves and roots (5 g) were extracted by percolation (96 h) with ethyl acetate and the residual solvent was removed under reduced pressure.

### 4.4. Volatile Concentrates Analysis

An aliquot (1 μL) of organic fraction of each sample was submitted for analysis of volatile components. The analyses were carried on a GC-MS Thermo Focus DSQ II (Thermo Fisher Scientific, Austin, TX, USA), under the following conditions: DB-5ms (30 m × 0.25 mm; 0.25 mm film thickness, Agilent, Santa Clara, CA, USA) fused-silica capillary column; programmed temperature, 60–240 °C (3 °C/min); injector temperature, 250 °C; carrier gas, helium, adjusted to a linear velocity of 32 cm/s (measured at 100 °C); injection type, split (2 μL); split flow was adjusted to yield 20:1 ratio; septum sweep was a constant 10 mL/min; EIMS (Electron Impact Mass Spectrometry), electron energy, 70 eV; temperature of the ion source and connection parts, 200 °C. The quantitative data regarding the volatile constituents were obtained by peak area normalization using a FOCUS GC/FID (Thermo Fisher Scientific, Austin, TX, USA) operated under similar conditions of the GC-MS, except the carrier gas, which was nitrogen. The retention indices were calculated for all the volatile constituents using a homologous series of *n*-alkanes (C8–C32, Sigma-Aldrich, St. Louis, MO, USA) [56].

### 4.5. Total Phenolics Content

The amount of total phenolics (TP) of the ethyl acetate extract was determined using the Folin-Ciocalteu procedure (Sigma-Aldrich, St. Louis, MO, USA) [57,58]. The extracts were dissolved in methanol at an initial concentration of 40 mg/mL and then diluted in water. Aliquots (500 μL) of the aqueous solution was mixed with 250 μL of Folin-Ciocalteu reagent (1.0 N, Sigma-Aldrich, St. Louis, MO, USA) and 1250 μL of sodium carbonate (75 g/L). The absorbance was measured after 30 min, at 760 nm and 25 °C (UV-Vis spectrophotometer, Biosystems RA2708, Costa Brava, Barcelona, Spain). The experimental calibration curve was prepared using gallic acid at concentrations of zero, 0.5, 1, 2, 4, 6, 8 and 10 mg/L, which were submitted to the same procedure. The total phenolics content are expressed as gallic acid equivalents (GAE) in milligrams per gram of extract (mg GAE/g).

### 4.6. Extract Samples Analysis

The ethyl acetate extracts were analyzed by a liquid phase chromatograph coupled to a mass spectrometer detector (HPLC-ESI-MS, Shimadzu, Tokyo, Japan), which was equipped with analytical pumps LC-20AD (HPLC-ESI-MS, Shimadzu, Tokyo, Japan) (2), sampler SIL-20AHT (HPLC-ESI-MS, Shimadzu, Tokyo, Japan), detector UV/Vis SPD-20A (HPLC-ESI-MS, Shimadzu, Tokyo, Japan), oven CTO-20A (HPLC-ESI-MS, Shimadzu, Tokyo, Japan) and controller CBM-20A (HPLC-ESI-MS, Shimadzu, Tokyo, Japan). The mobile phase was methanol (MeOH) (plus 0.1% formic acid) and water (plus 0.1% formic acid) in a gradient of 30% of MeOH maintained for 2 min, then, from 2 to 25 min the percentage of MeOH was increased to 100% and kept for an additional 5 min. The column was Luna 5u PFP (2) 100 A, 150 × 2 mm (Phenomenex, Torrance, CA, USA). The UV detector was kept in 254 and 280 nm, the oven was in 40 °C. The flow was 200 µL/min and it was injected directly in the mass spectrometer. The mass spectrometer Bruker micrOTOF-QII (Bruker, Billerica, MA, USA) was in positive mode with nebulizer and dry gas (N_2_, 8 L/min), at 4 Bar. The dry temperature was 200 °C and the collision and quadrupole energy was 12 and 6 eV, respectively. The funnels RF1 and RF2 were set to 200 Vpp.

### 4.7. Statistical Analysis

Samples were assayed in triplicate in phenolic content assay and the results are shown as means ± standard deviation. Analysis of variance was conducted and the differences between variables were tested for significance by one-way analysis of variance (ANOVA) with Tukey’s post hoc test using GraphPad version 5.0. (GraphPad Software, San Diego, CA, USA) Differences at *p* < 0.05 were considered statistically significant. The identified volatile compounds were used as variables in the Principal Component Analysis (PCA) to classify and group the inoculated and control plants. These data were analyzed using the Minitab software (free version, Minitab software, State College, PA, USA).

## Figures and Tables

**Table 1:** Volatile compounds (%) of the leaves of Bragantina cultivar: inoculated plants (IP) and control plants (CP).

Bragantina Cultivar			7 dpi		15 dpi		21 dpi		30 dpi		45 dpi	
**Constituents**	**RI^a^**	**RI^b^**	**IP**	**CP**	**IP**	**CP**	**IP**	**CP**	**IP**	**CP**	**IP**	**CP**
3*E*-Hexenol	845	844	0.3	0.5	0.3 ^+^	0.1	0.6 *		0.7 *		0.2 *	
Limonene	1022	1024	0.1	0.1		0.1					0.1	0.1
*E*-β-Ocimene	1029	1044	1.2 ^+^	0.1		0.5	1.1	0.8			1.0 ^+^	0.5
Linalool	1091	1095	0.5	0.7	0.3	0.4	0.4	0.3			0.3 ^+^	0.1
Pregeijerene	1269	1285	0.3	0.5	0.2	0.2		0.3			0.2	0.2
2-Undecanone	1285	1293	0.1 *		0.1	0.1					0.1	0.1
δ-Elemene	1325	1335		0.1	0.1	0.1		0.1				0.1
α-Ylangene	1359	1373	0.1 *		0.1	0.1						0.1
β-Bourbonene	1374	1387	0.1 *									
β-Elemene	1380	1389	0.2 ^+^	0.1	0.2	0.2						0.1
β-Caryophyllene	1407	1417	1.0 ^+^	0.4	1.1	1.2	0.7	0.9	0.9	0.9	0.6	0.8
γ-Elemene	1418	1434	0.1	0.1	0.2 ^+^	0.1						0.1
Geranyl acetone	1440	1453	0.1	0.1	0.1	0.1					0.1 *	
*E*-β-Farnesene	1443	1454	0.7 ^+^	0.2	0.8	0.8	0.7	0.7			0.7	0.5
Germacrene D	1470	1484	4.2 ^+^	1.4	5.7	5.1	3.4	4.3	2.2 ^−^	6.9	2.5	4.1
β-Selinene	1476	1489	0.1	0.1	0.1 ^−^	0.2			0.5 *		0.2	0.2
α-Zingiberene	1483	1493	0.2 ^+^	0.1	0.2 ^−^	0.4		0.2	0.9 *		0.9	0.7
α-Muurolene	1490	1500	0.1 *		0.1	0.1			0.4 *		0.2 ^+^	0.1
β-Bisabolene	1497	1505	3.6 *	1.1	4.3	4.1	4.2	4.2	6.0 ^+^	3.6	3.0	2.8
β-Sesquiphellandrene	1514	1521	0.1 *		0.3	0.4			0.7 *		0.6	0.5
*E*-γ-Bisabolene	1517	1529	0.1	0.1						2.0		
Elemol	1535	1548	19.0	27.3	24.4	22.3	15.3	28.4	0.7 ^−^	32.9	3.4 ^−^	9.2
Germacrene B	1545	1559	0.3 *					0.3			0.1	0.1
*E*-Nerolidol	1551	1561	1.8	1.7	1.5	2.0	1.7	1.2		1.1	1.2	1.0
Caryophyllene oxide	1569	1582	0.2	0.2	0.1 *							0.1
Longiborneol	1591	1599	0.1	0.1	0.1 *						0.1	0.1
β-Eudesmol	1640	1649	0.3	0.3		0.3		0.3	10.7 *		14.9	12.2
α-Bisabolol	1679	1685	51.4	53.9	44.8	47.9	64.3	51.4	60.1	49.8	60.7	54.6
(2*Z*,6*Z*)-Farnesol	1696	1698	1.6 *		1.9	1.5		0.5				1.5
(2*Z*,6*E*)-Farnesol	1704	1722	3.2 *		3.5	3.2		1.1		1.5		2.5
Monoterpene hydrocarbons			1.4	0.3	0.1	0.7	1.1	0.8			1.2	0.6
Oxygenated monoterpenes			0.9	1.2	0.6	0.7	0.4	0.6			0.6	0.4
Sesquiterpene hydrocarbons			10.9	3.7	13.2	12.8	9.0	10.7	11.6	13.4	8.8	10.2
Oxygenated sesquiterpenes			77.6	83.5	76.3	77.2	81.3	82.9	71.5	85.3	80.3	81.2
Others			0.3	0.5	0.3	0.1	0.6		0.7		0.2	
Total			91.1	89.2	90.5	91.5	92.4	95.0	83.8	98.7	91.1	92.4

RI^a^: Retention index calculated; RI^b^: Retention Index of Literature [17]; * Compounds produced only by inoculated plants; ^+^ Compounds that increased more than 50% in inoculated plants, ^−^ Compounds that reduced more than 50% in inoculated plants.

**Table 2:** Volatile compounds (%) of the leaves of Cingapura cultivar: inoculated plants (IP) and control plants (CP).

Cingapura Cultivar			7 dpi		15 dpi		21 dpi		30 dpi		45 dpi	
**Constituents**	**RI^a^**	**RI^b^**	**IP**	**CP**	**IP**	**CP**	**IP**	**CP**	**IP**	**CP**	**IP**	**CP**
3*E*-Hexenol	846	844	1.6 *		0.3 *						0.2 ^+^	0.1
α-Pinene	928	932	0.5 *		0.1	0.1						
β-Pinene	970	974				0.1						
Myrcene	983	988				0.1						
δ-2-Carene	1004	1001	0.2 *		0.1 *							
Limonene	1023	1024	0.2 *									
*E*-β-Ocimene	1040	1044	0.4 *		0.2	0.2						
Linalool	1092	1095	2.6	2.0	1.0	1.4	1.2 ^−^	2.5	1.5 ^+^	0.6	0.5	0.8
Methyl citronellate	1250	1257				0.1						
Carvenone	1252	1255	0.1 *		0.1	0.1					0.1	0.1
2-Undecanone	1285	1293				0.1						
δ-Elemene	1322	1335	45.9	57.6	73.6 ^+^	48.7	48.8	55.1	61.2	57.1	55.8	48.1
δ-Cubebene	1337								0.6 *			
α-Cubebene	1365	1345	0.4 ^−^	2.1	0.4	0.3	0.6 *			0.5	0.9	0.7
α-Copaene	1365	1374							1.0 *			
α-Ylangene	1367	1373	1.2 *		0.8 ^+^	0.4	1.1 *			1.2	1.2	1.4
β-Elemene	1379	1389	2.7	4.1	2.1	1.6	3.1	3.0	3.2	2.7	3.1	3.9
α-Gurjunene	1396	1409	0.7 *		0.4	0.3	0.8		0.8	0.7	1.0	1.1
β-Caryophyllene	1407	1417	3.0	3.8	2.3	3.1	6.6	8.7	3.9	3.0	5.4	4.9
6,9-Guaiadiene	1430	1442	0.1 *		0.5 *					0.7		1.2
α-Humulene	1443	1452	0.6 ^−^	2.2	0.2	0.5	0.9 *		0.8 *		0.9 *	
Thujopsadiene	1449	1465	0.3 *			0.3	0.5 *		0.4 *		0.3 *	
allo-Aromadendrene	1461	1458	0.1 *		0.1 *		0.5 *				0.4 *	
9-*epi*-*E*-Caryophyllene	1465	1464	0.2 *		0.1 ^−^	0.2						0.4
Germacrene D	1470	1484	0.6 *		0.6 ^+^	0.3	0.6 *		0.7	0.6	0.5	0.7
β-Selinene	1476	1489	2.2	4.2	1.8	1.3	3.3	2.9	3.4	2.5	3.6	4.4
α-Zingiberene	1482	1493	2.9 ^−^	6.9	2.9	2.3	5.2	5.0	5.2	4.3	5.5	6.8
*Z*- Cycloisolongifol-5-ol	1498	1513				0.3						
*epi*-Cubebol	1503	1493	0.3 *		0.1 ^−^	0.7	0.9 *				0.3	0.4
δ-Amorphene	1507	1511	0.7 *		0.3 *				0.7 ^+^	0.4	0.4	0.7
γ-Vetivenene	1534	1531	0.2 *			0.1						0.1
γ-Elemene	1545	1465				0.1						
*E*-Nerolidol	1549	1561	2.9	4.8	1.3 ^−^	8.5	3.9	6.6	2.7	4.0	2.7	2.2
Germacrene D-4-ol	1564	1574	0.2 *		0.1	0.3	0.3 *			0.4	0.4	0.5
Caryophyllene oxide	1576	1582	6.6	6.3	2.9	3.9					3.6	3.8
Spathulenol	1577	1577				3.1	6.1	6.1	3.6	4.5	0.3 *	
β-Copaen-4α-ol	1595	1590				2.8	1.5 *		1.7	1.7	0.8 *	
β-Atlantol	1596	1608	3.4 *									
Ledol	1602	1602				0.4						
1,10-di-*epi*-Cubenol	1607	1618	1.6 *		0.6 ^−^	1.2	1.0 *		0.8	1.3		1.1
Muurola-4,10(14)-dien-1β-ol	1613	1630	4.3	5.9	2.0 ^−^	4.9	6.5	5.5	3.7	5.3	3.8	3.1
α-Cadinol	1642	1652	0.5 *		0.2 ^−^	0.4	0.4 *		0.3 *		0.3	0.5
*allo*-Aromadendrene epoxide	1649	1639	0.2 *		0.1 *						0.1 ^−^	0.2
Khusinol	1659	1679	0.7 *		0.3 ^−^	0.6			0.4 *		0.2	0.5
Germacra-4(15),5,10(14)-trien-1α-ol	1672	1685	0.2 *		0.1	0.3						0.1
2*Z*,6*Z*-Farnesal	1684	1684	0.2 *		0.1 *						0.1	0.1
2*E*, 6*Z*-Farnesal	1696	1713				0.6						
2*Z*,6*E*-Farnesal	1696	1715					0.2 *		0.4 *			
Eudesma-4,11-dien-2α-ol	1704	1704	1.2 *		0.6 ^−^	1.9	0.9 *		1.2	1.1	1.1 ^−^	2.2
*E*, *E*, *E*-7,10,13-Hexadecatrienal	1718	1824					0.8 *					
*iso*-Aromadendrene epoxide	1719	1719	0.6 *		0.3 ^−^	0.6			0.4	0.5	0.3	0.5
2*E*,6*E*-Farnesal	1725	1725	0.4 *			1.0			0.8	0.7	0.8 ^−^	2.0
Monoterpene hydrocarbons			1.3		0.4	0.7						
Oxygenated monoterpenes			2.7	2.0	1.1	1.7	1.2	2.5	1.5	0.6	0.6	0.9
Sesquiterpene hydrocarbons			61.8	80.6	86.1	59.5	72.0	74.7	81.9	73.7	79.0	74.4
Oxygenated sesquiterpenes			23.3	17.0	8.7	31.7	22.8	18.2	16.0	19.5	14.8	17.2
Others			1.6		0.3						0.2	0.1
Total			90.7	99.9	96.4	93.4	95.7	95.4	99.4	93.8	94.6	92.6

RI^a^: Retention index calculated; RI^b^: Retention Index of Literature [17]. * Compounds produced only by inoculated plants, ^+^ Compounds that increased more than 50% in inoculated plants, ^−^ Compounds that reduced more than 50% in inoculated plants.

**Table 3:** Chemical profile of root extracts of Bragantina cultivar: inoculated plants (IP) and control plants (CP).

RT	Alkamides	MW	MF	7 dpi		15 dpi		21 dpi		30 dpi		45 dpi	
**(min)**	**(Figure 4)**	**(Molecular Weight)**	**(Molecular Formula)**	**IP**	**CP**	**IP**	**CP**	**IP**	**CP**	**IP**	**CP**	**IP**	**CP**
17.1	1R	341.4402	C_21_H_27_NO_3_	X	X			−	X	−	X		
18.8	2aR/2bR	273.3243	C_16_H_19_NO_3_	+		X	X						
19.7	3aR/3bR	359.5827	C_24_H_41_NO					−	X	X	X		
20.5	4R	339.4246	C_21_H_25_NO_3_							X	X	−	X
21.4	5R	381.5035	C_24_H_31_NO_3_	X	X	X	X	X	X	X	X		
22.2	6R	285.3350	C_17_H_19_NO_3_	X	X	X	X	X	X	X	X	X	X
22.6	7R	452.8303	C_31_H_64_O					+					
23.3	8aR/8bR	224.3587	C_14_H_26_NO	X	X	X	X	X	X	X	X	X	X
23.8	9aR/9bR	333.5457	C_22_H_39_NO							X	X	+	
24.1	10R	311.3720	C_19_H_21_NO_3_									+	
25.6	11aR/11bR	361.5983	C_24_H_43_NO							−	X	X	X
26.2	12R	367.4772	C_23_H_29_NO_3_	X	X	X	X	X	X	X	X	X	X
26.8	13R	383.5191	C_24_H_33_NO_3_	X	X	X	X	X	X	X	X	X	X
27.7	9aR/9bR	333.5457	C_22_H_39_NO	X	X	+		−	X	X	X	X	X
28.3	14aR/14bR	335.5613	C_22_H_41_NO	X	X	X	X	X	X	X	X	X	X
29.0	15aR/15bR	363.6139	C_24_H_45_NO					−	X				

RT: Retention time; MW: Molecular weight; MF: Molecular formula; Alkamides in bold represent changes in function of the infection; (X): Alkamides present in inoculated and control plants; (−) Alkamides present in control plants and absent in inoculated plants; (+) Alkamide present only in inoculated plants.

**Table 4:** Chemical profile of root extracts of Cingapura cultivar: inoculated plants (IP) and control plants (CP).

RT	Alkamides	MW	MF	7 dpi		15 dpi		21 dpi		30 dpi		45 dpi	
**(min)**	**(Figure 4)**	**(Molecular Weight)**	**(Molecular Formula)**	**IP**	**CP**	**IP**	**CP**	**IP**	**CP**	**IP**	**CP**	**IP**	**CP**
22.2	6R	285.3350	C_17_H_19_NO_3_	X	X	X	X	X	X	−	X	X	X
23.3	8aR/8bR	224.3587	C_14_H_26_NO	X	X	X	X	X	X	X	X	X	X
23.9	9aR/9bR	333.5457	C_22_H_39_NO	X	X	−	X	X	X	−	X	X	X
24.0	14aR/14bR	335.5613	C_22_H_41_NO	+				X	X	−	X	X	X
24.1	9aR/9bR	333.5457	C_22_H_39_NO	X	X			X	X	−	X	X	X
24.4	16R	235.3616	C_15_H_25_NO	X	X	X	X	X	X	−	X	X	X
26.3	12R	367.4772	C_23_H_29_NO_3_	X	X	X	X	X	X	X	X	X	X
26.8	13R	383.5191	C_24_H_33_NO_3_	X	X	X	X	X	X	X	X	X	X

RT: Retention time; MW: Molecular weight; MF: Molecular formula; Alkamides in bold represent changes in function of the infection; (X): Alkamides present in inoculated plants and control plants; (−): Alkamides present in control plants and absent in inoculated plants; (+): Alkamides present only in inoculated plants.
